# Open Versus Percutaneous Stabilization of Thoracolumbar Fractures: A Large Retrospective Analysis of Safety and Reoperation Rates

**DOI:** 10.7759/cureus.61369

**Published:** 2024-05-30

**Authors:** David R Peters, Tripp Owen, Ummey Hani, Deborah Pfortmiller, Christopher Holland, Domagoj Coric, Michael Bohl, Paul K Kim

**Affiliations:** 1 Neurosurgery, Atrium Health Carolinas Medical Center, Charlotte, USA; 2 Neurosurgery, Carolina Neurosurgery & Spine Associates, Charlotte, USA; 3 Neurosurgery/Statistics, Carolina Neurosurgery & Spine Associates, Charlotte, USA

**Keywords:** spine trauma, open stabilization, percutaneous stabilization, lumbar spine fracture, thoracic spine fracture

## Abstract

Background: Thoracolumbar fractures (TLF) requiring surgical intervention can be treated with either open or percutaneous stabilization, each with some distinct risks and benefits. There is insufficient evidence available to support one approach as superior.

Methods: Patients who underwent spinal fixation for TLF between 2008 and 2020 were reviewed. Patients with one or two levels of fracture treated with either open or percutaneous stabilization were included. Exclusion criteria were more than two levels of fracture, patients requiring corpectomy, stabilization constructs that crossed the cervicothoracic or lumbosacral junction, history of previous thoracolumbar fusion at the same level, spinal neoplasm, anterior or lateral fixation, and spinal infection. Demographic, operative, and clinical data were collected for all patients.

Results: 691 patients (377 open, 314 percutaneous) met the inclusion criteria. Patients in the percutaneous cohort sustained lower estimated blood loss (73 vs 334 ml; p< 0.001) and shorter length of surgery (114 vs. 151 minutes; p< 0.001). No differences were observed in the length of hospital stay or overall reoperation rates. Asymptomatic (7.0% vs 0.8%) and symptomatic (3.5% vs 0.5%) hardware removal was more common with the percutaneous cohort, while the incidence of revision surgery due to hardware failure requiring the extension of the construct (1.9% vs 5.8%) and infection (1.9% vs 6.4%) was greater in the open group.

Conclusion: Percutaneous stabilization for TLF was associated with shorter operative time, less blood loss, lower infection rate, higher rates of elective hardware removal, and lower rates of hardware failure requiring extension of the construct compared to open stabilization.

## Introduction

Thoracolumbar fractures (TLFs) are the most frequent fractures of the spinal column, accounting for around 70% of all traumatic spinal injuries [1,2]. While many may be treated non-surgically, others with neurological injury and instability require operative intervention to reduce the risk of spinal deformity, pain, and permanent neurological deficit [3]. Historically, the intervention of choice has been open surgery with stabilization and fusion, along with decompression if necessary. Open techniques involve a large midline incision and dissection of the paraspinal muscles laterally off the posterior bony elements of the spine. Screws and rods are then placed under direct visualization. This has demonstrated good stability, correction of spinal alignment, and direct decompression of the spinal canal. However, significant perioperative complications exist, including blood loss and infection [4,5]. In addition, any long-segment fusion introduces the long-term risk of adjacent segment degeneration [6,7].

To reduce the negative consequences of open stabilization, percutaneous methods of spinal fixation were developed over 20 years ago [8,9] and soon after applied to spinal trauma [10]. Percutaneous stabilization typically involves multiple small incisions with placement of the screws and rods using image guidance. The screws are placed through the paraspinal muscles since the muscles are not elevated off the posterior spine. Short-term benefits of percutaneous stabilization include reduced blood loss, reduced postoperative pain, and faster return to previous activity [11-14]. Potential limitations include reduced ability to correct deformity and lower rates of fusion [14], but high-quality long-term outcome data are still lacking. Current guidelines state that both open and percutaneous techniques may be considered for the stabilization of TLFs as there is insufficient evidence to recommend one method over the other [15]. Here, we conducted the largest known comparison of safety and reoperation rates between open and percutaneous stabilization of TLF.

## Materials and methods

This was a retrospective study conducted at the Atrium Health Carolinas Medical Center, Charlotte, North Carolina, United States. All patients with TLFs treated operatively between November 25, 2008 (the date of first percutaneous stabilization at our institution) and December 31, 2020, were retrospectively reviewed. Inclusion criteria included all spine trauma patients with one or two levels of fracture of the thoracolumbar spine treated with either open or percutaneous posterior pedicle screw stabilization. Exclusion criteria included more than two levels of fracture, injuries requiring corpectomy, stabilization constructs that crossed the cervicothoracic junction or lumbosacral junction, history of previous thoracolumbar fusion at the same level, spinal neoplasm, anterior or lateral fixation, and spinal infection. Patients gave consent for all procedures after discussion with the primary surgeon at the time of the procedure. Clinical information was retrospectively collected and analyzed.

Demographic, operative, and clinical data were collected for all patients. Operative notes were reviewed to determine if the patient was treated with open or percutaneous stabilization, to record blood loss, number of screws used, presence of fusion graft, decompression, and length of surgery. Open stabilization involved all procedures where a midline incision with an elevation of the paraspinal muscles off the posterior spinal bony elements was performed prior to pedicle screw placement. Percutaneous stabilization included all procedures where the midline paraspinal muscles were not elevated, as screws were placed through paramedian, transmuscular windows under image guidance. Decompression in the percutaneous group was performed using a tubular system and involved laminectomy at the level(s) of stenosis.

A subgroup analysis was also performed which excluded all patients in both groups who had undergone intraoperative decompression. All statistical analyses were performed using IBM SPSS Statistics for Windows, Version 27.0 (IBM Corp., Armonk, New York, United States). Univariable parametric data were provided in mean ± standard deviation (SD) and nonparametric data were provided as a percentage frequency. Bivariate analyses were conducted using independent t-tests and chi-square tests. Statistical significance level was set at p <.05.

## Results

Patient demographics and intraoperative device utilization

A total of 691 patients (377 open, 314 percutaneous) met the inclusion criteria. Table 1 shows the baseline clinical characteristics and intraoperative device utilization for the patients in each cohort. No significant differences existed for patient age, body mass index (BMI), and number of levels of fracture between the cohorts. The group that underwent percutaneous screw placement had a significantly greater proportion of males (67% vs. 56%; p=.003). Patients in the percutaneous cohort required fewer total screws (6.95 vs. 8.10; p< 0.001), required decompression less frequently (8.0% vs. 70.3%; p< 0.001), and were less likely to undergo fusion grafting (18.2% vs. 96.0%; p< 0.001).

**Table 1:** Patient demographics and intraoperative device utilization

	Open Stabilization Group	Percutaneous Stabilization Group	t/c^2^	p-value
Number of patients, n (%)	377 (54.6%)	314 (45.4%)		
Age (years), mean ±SD	52.14 ±19.9	50.9 ±20.8	-0.78	.218
BMI (*kg/m^2^*), mean ±SD	28.16 ±7.5	28.8 ±8.3	1.08	.140
Male:Female, n (%)	211:166 (56%:44%)	210:104 (67%:33%)	8.57	.003
Fracture Levels, n (%)				
1	265 (70.3%)	230 (73.2%)	0.74	.391
2	112 (29.7%)	84 (26.8%)		
Total screws, mean±SD	8.10 ±2.73	6.95 ±2.14	-6.08	< .001>
Decompression, n (%)	265 (70.3%)	25 (8.0%)	273.3	< .001>
Fusion Graft, n (%)	362 (96.0%)	57 (18.2%)	435.2	< .001>
Imaging, n (%)				
Fluoroscopic	256 (67.9%)	190 (60.5%)	4.09	.043
O-arm	121 (32.1%)	124 (39.5%)		

Perioperative facility utilization, safety, and outcomes

Table 2 shows the safety and facility utilization by the patients in each cohort. The percutaneous cohort sustained lower estimated blood loss (73 vs 334 ml; p< 0.001) and shorter length of surgery (114 vs. 151 minutes; p< 0.001). No significant differences were observed in the length of hospital stay or overall reoperation rates between both groups. A significantly greater frequency of elective and symptomatic hardware removal was seen with the percutaneous cohort, while the incidence of infection and hardware failure was significantly greater in the open group (p< 0.01). Hardware failure was defined as a repeat operation that required an extension of the original construct. Patients in the open group had longer mean follow-up (12.6 vs 9.5 months, p=.009), but there was no significant difference in the proportion lost to follow-up after hospital discharge.

**Table 2:** Outcomes and safety

Characteristics	Open Stabilization Group (N=377)	Percutaneous Stabilization Group (N=314)	t/c^2^	p-value
Length of surgery (minutes), mean ±SD	151 ± 56	114 ± 59	9.13	< .001>
Estimated blood loss (mL), mean±SD	334 ± 329	73 ± 72	-15.86	< .001>
Length of stay (days), mean±SD	10.4 ± 12.2	9.5 ±11.7	-0.99	.161
Reoperation rate, n (%)	59 (15.6%)	50 (15.9%)	0.01	.922
Reason for Reoperation, n (%)				
Asymptomatic hardware removal	3 (0.8%)	22 (7.0%)	18.95	< .001>
Symptomatic hardware removal	2 (0.5%)	11 (3.5%)	8.20	.004
Hardware failure	22 (5.8%)	6 (1.9%)	6.79	.009
Wound infection	24 (6.4%)	6 (1.9%)	8.19	.004
Perioperative Reoperation, n (%)				
Cerebrospinal fluid leak	4 (1.1%)	0 (0%)	1.38	.711
Screw repositioning	1 (0.3%)	1 (0.3%)		
Epidural hematoma	3 (0.8%)	4 (1.3%)		
Follow-up				
No follow-up after discharge, n (%)	70 (18.6%)	52 (16.5%)	0.475	.491
Mean, median, range (months)	12.6, 13, 1:66	9.5, 10, 1:117	-2.36	.009

Table 3 shows the safety and facility utilization for the subgroup analysis where patients in each group undergoing decompression were excluded. The percutaneous cohort sustained lower estimated blood loss (68 vs 237 ml; p< 0.001) and shorter length of surgery (108 vs. 127 minutes; p< 0.001). No significant differences were observed in the length of hospital stay or overall reoperation rates between both groups. A significantly greater frequency of asymptomatic hardware removal was seen with the percutaneous cohort, while the incidence of infection was significantly greater in the open group (p< 0.05). The frequency of asymptomatic hardware removal was higher for the percutaneous group, while the frequency of hardware failure was higher for the open group, although these two comparisons between groups did not reach a level of significance. There was no significant difference in mean follow up (9.7 vs 9.2 months, p=.414), or in the proportion of patients lost to follow-up after hospital discharge.

**Table 3:** Outcomes and safety, subgroup analysis with no decompression N/A: not available

Characteristics	Open Stabilization Group (N=112)	Percutaneous Stabilization Group (N=289)	t/c^2^	p-value
Length of surgery (minutes), mean±SD	127 ± 43	108 ± 57	8.45	< .001>
Estimated blood loss (mL), mean±SD	237 ± 205	68 ± 65	-12.08	< .001>
Standard deviation (days), mean±SD	11.4 ± 11.3	9.4 ±11.7	-1.57	.058
Reoperation rate, n (%)	13 (11.6%)	46 (15.9%)	1.19	.274
Reason for reoperation, n (%)				
Asymptomatic hardware removal	2 (1.8%)	21 (7.3%)	4.48	.034
Symptomatic hardware removal	1 (0.9%)	10 (3.4%)	1.99	.158
Hardware failure	3 (2.7%)	6 (2.1%)	0.13	.715
Wound infection	7 (6.3%)	6 (2.1%)	4.48	.034
Perioperative reoperation n (%)				
Cerebrospinal fluid leak	0 (0%)	0 (0%)	N/A	N/A
Screw repositioning	0 (0%)	1 (0.3%)		
Epidural hematoma	0 (0%)	2 (0.7%)		
Follow-up				
No follow-up after discharge, n (%)	23 (20.5%)	49 (16.9%)	0.70	.402
Mean, Median, Range (months)	9.7, 10, 1:51	9.2, 10, 1:117	0.22	.414

## Discussion

Surgical treatment of thoracolumbar fractures typically involves the placement of pedicle screws and rods to stabilize the spine and prevent further deformity, spinal cord, or nerve damage. This procedure can be grouped into two general approaches: open and percutaneous. Open stabilization has been the historical standard of care. There are well-known risks associated with this treatment. The infection rate for an open posterior approach with arthrodesis varies from 3.1% to 10% [4,5]. We report an infection rate of 6.4% for open treatment in our cohort, in line with previous reports. Additionally, paraspinal denervation and ischemia may occur due to coagulation of the paraspinal blood vessels, increased muscle pressure from retractor use, and extensive dissection of the lateral muscles during a standard posterior approach [16-18]. The extensive dissection required for open approaches may increase the risk of construct failure, particularly in patients with diminished bone quality. Denervation, ischemia, and re-vascularization injury of paraspinal muscles leads to atrophy, scarring, and weakness [17,19].

In response to this problem, percutaneous spinal fixation systems were designed over 20 years to reduce injury to the paraspinal soft tissues [8-10]. The short-term benefits of percutaneous over open surgery are relatively well defined and include less tissue damage, reduced risk of infection, less blood loss, shorter surgery time, less post-operative pain, and a faster return to previous activity [11-14,20-22]. Consistent with previous reports, our data shows a significantly lower infection rate (1.9% vs 6.4%, p = .004), shorter surgical time (114 minutes vs 151 minutes, p < .001), and lower blood loss (73 mL vs 334 mL, p < .001) in percutaneous stabilization. These advantages are relevant in the trauma population, who often have additional concurrent traumatic injuries and acute blood loss anemia. Despite the logical and well-known short-term benefits, there remains insufficient high-quality, prospective data with long-term outcomes available to recommend percutaneous fixation over open surgical fixation, or vice versa, for thoracolumbar fractures [15].

One of the biggest concerns around the outcomes of percutaneous stabilization relates to its reduced ability for arthrodesis compared to open techniques, and, thus, potentially a higher rate of construct failure. Additionally, a minimally invasive tube can be utilized for decompression and/or to deliver bone graft for fusion, but it is unclear how the quality of decompression and the fusion rate compare to an open technique [23]. Open surgery allows for much better preparation of bone graft and fusion sites as well as direct visualization of the decompression. Logically, open surgery should be expected to have better decompression and much better rates of fusion than percutaneous approaches, although the extent and clinical relevance of this benefit are not well defined.

Arthrodesis has been commonly utilized to safeguard against hardware failure that is caused by spinal mobility around the fixation system. The prevailing belief has been that without the permanent stability offered by arthrodesis, the fixation system would inevitably fail and result in a painful and potentially unstable condition that often requires reoperation. On the other hand, arthrodesis sacrifices spinal mobility and increases the risk of adjacent segment disease [6,7]. Several recent studies have challenged this widely accepted belief regarding arthrodesis, presenting data suggesting that fusion is not necessary in the treatment of TLFs [24-27]. Isolated bone fractures without significant disc space or ligamentous involvement may be more amenable to stabilization without arthrodesis. Furthermore, there is minimal data regarding the rate of pseudarthrosis requiring reoperation for percutaneous stabilization of TLFs.

To preserve the motion of the spine, two surgeons in our study chose routine prophylactic removal of the percutaneous construct in young patients, once the fracture healing was confirmed radiographically. This is reflected in the 22 patients (7.0%) in the percutaneous cohort who underwent asymptomatic hardware removal. Eleven other patients (3.5%) developed pain after healing of the fracture that was attributed to the instrumentation, and these patients were treated with hardware removal. It is important to note that 57 (18.2%) patients in the percutaneous group were treated by a technique that included decortication of the facets and placement of bone graft through a minimally invasive tube into and around the facet joints.

While the overall rates of reoperation were similar in the two cohorts (15.6% open, 15.9% percutaneous, p=.922), the indication for reoperation was significantly different. The open group had higher rates of infection and hardware failure requiring revision with a longer fixation construct. The percutaneous group had higher rates of asymptomatic and symptomatic hardware removal. Rates of immediate postoperative complications including cerebrospinal fluid (CSF) leak, epidural hematoma, and misplaced screws were similar. At our institution, the procedure for hardware removal is usually performed as an outpatient surgery, while treatment for extension of the construct or wound infection typically requires a prolonged inpatient hospitalization.

The difference in the rate of decompression between the two groups is an obvious confounding factor in our initial comparison. Thus, we removed this confounding factor for our subgroup analysis. After removing all instances of decompression in both groups, our results remained similar to the overall cohort. While overall reoperation rates were similar, there was still a significantly increased infection rate and lower asymptomatic hardware removal rate in the open group. There was a trend towards a higher rate of hardware failure and a lower rate of symptomatic hardware removal in the open group, but this did not reach a level of significance. There were no perioperative complications in the open group that did not undergo decompression.

Our data suggests several things. First, in line with previously published data, our series shows that percutaneous stabilization has less blood loss, faster surgery time, and lower rates of infection compared to open stabilization. Second, the rate of symptomatic hardware removal in percutaneous fixation is low, and it is not clear that elective asymptomatic hardware removal should be routinely performed. Prospective, randomized trials are needed to further investigate this. Third, open treatment has lower rates of both asymptomatic and symptomatic hardware removal. Perioperative complications are similar between the two groups.

There are several limitations to this study. First, retrospective studies cannot definitively establish one treatment as superior to another. Both open and percutaneous approaches, as they were chosen for these patients in this institution, appear to be safe and reasonable treatments. Second, long-term outcome data on trauma patients is often difficult to obtain. In our study, around 17% of patients in each cohort did not have any follow-up after hospital discharge. While our follow-up approaching one year is not adequate for long-term instrumented fusion constructs, extended follow-up in a trauma patient population is challenging. If the fracture has healed and the patient is clinically doing well without complaints, our institution does not routinely follow up on this cohort of patients beyond one year. Reoperation rate was the best outcome measure available since patient-recorded outcome measures including pain and quality of life as well as radiographic outcomes are not available. An additional limitation to this study is that percutaneous stabilization may be preferentially used for more stable, less complex fractures due to a reduced ability for fusion, decompression, and spinal realignment compared to open techniques [28]. We tried to partially correct for this with our subgroup analysis but it obviously does not eliminate all confounding factors. It is certainly possible that the increased incidence of hardware failure and infection seen in our study is due to the presence of more complex fractures in the open cohort. The superiority of open or percutaneous treatment for thoracolumbar fractures can only be clarified by prospective, randomized trials with long-term follow-up.

## Conclusions

Percutaneous stabilization for TLF was associated with shorter operative time, less blood loss, and a lower infection rate compared to open stabilization. Perioperative complications are similar between the two groups. Overall re-operation rates were similar, but percutaneous fixation had higher rates of hardware removal, most of which was asymptomatic, prophylactic removal. The rate of symptomatic hardware removal after percutaneous fixation was only 3.5%, suggesting that asymptomatic removal may not be necessary. Prospective, randomized trials with long-term follow-up are needed to better define the benefit and risk of percutaneous vs open stabilization of thoracolumbar fractures.
